# Books and Magazines

**Published:** 1911-07

**Authors:** 


### Books and Magazines.

Gonorrhea in the Male—A Practical Guide to Its Treatment. By Abr. L. Wolbarst, M. D., New York. The International Journal of Surgery Co., New York. 1911. Price, not stated.

This is a small book, but one of much value. Most doctors regard gonorrhea as a comparativley harmless disease, and with young men it is a joke. The great danger of systemic infection and its far-reaching destructive effect upon the wife and mother and offspring constitute one of the most potent factors in the swift decay of the race. It is the most important feeder of the woman's hospitals and the gynecologist's table. The practical value of this little book can hardly be overestimated. Gonorrhea is more than a specific urethritis.

---

Clinical Treatise on Inebriety. By T. D. Crothers, M. D., Superintendent Walnut Lodge Hospital, Hartford, Conn., Editor of the *Journal of Inebriety,* Author of Morphinism, and Narcomania, Drug Habits and their Treatment, etc.; Recording Secretary of the American Medical Society for the Study of Alcohol and Other Narcotics; Member of the American Medical Association, the British Medical Association, Honorable Member of the British Society for the Study of Inebriety, etc. Price, $3.00, express prepaid. Harvey Publishing Co., Medical Book Publishers, Merchants' Building, Cincinnati, Ohio.

This is the first scientific work and study of the great alcoholic problem by an American writer in which the subject is treated entirely from its scientific side, regardless of opinions or theories, but based on facts that have been gathered from long experience and careful examination.

The author, Dr. T. D. Crothers, is without doubt the most widely known medical man as a student of this subject in America. His practical experience, extending over thirty-five years as the manager of a hospital for inebriates and a scientific student of this subject, gives the assurance of a very broad and practical knowledge of the alcoholic problem.

We are confident that this book will open a new field of inquiry and practice for the medical man and materially aid in the promotion of a thorough scientific study of the facts, which so far have been largely in the hands of irregulars and persons unacquainted with the significance and meaning of this great psychological and hygienic problem of the age.

---

COMPEND OF THE PRACTICE OF MEDICINE.—Giving the Synonyms, Definition, Causes, Symptoms, Pathology, Prognosis, Diagnosis, Treatment, etc., of each Disease, Including a Section on Mental Diseases and one on Diseases of the Skin. By Daniel E. Hughes, M. D., Late Chief Resident Physician, Philadelphia Hospital, formerly Demonstrator of Clinical Medicine, Jefferson Medical College, Philadelphia. P. Blakiston's Son & Co., Philadelphia, Publishers. Tenth Edition. Thoroughly revised and enlarged by R. J. E. Scott, M. A., B. C. L., M. D., Attending Physician to the Demilt Dispensary, New York, author of the "State Board Examination Series," etc. With 63 Illustrations. 12mo; XVIII—878 pages. The Leather-bound Series of Manuals. Full Limp Leather, Gilt Edges, Round Corners, $2.50.

There are books which have met with such a large measure of success that the publication of a new edition seems to call for more than a mere mention of the fact of reissue.

Of Hughes' "Practice" we have now published ten editions, averaging 5000 copies, a sale which has been exceeded by very few books on any branch of medicine.

It may be taken as self-evident that no book on any subject whatsoever could have so wide a distribution unless it had intrinsic merit. It must be, if a novel of extreme interest, if on history, both interesting and true, but if on a scientific subject, it must be practical, up to date, useful. No amount of advertising will result in such success unless back of it is a book which physicians and students find helpful, and Hughes is all of this.

This tenth edition has been thoroughly revised, many changes and additions were necessary, and there has been some rearrangement of the subject matter in order to adapt it to modern classification. Several entirely new sections and numerous tables of differential diagnosis have been added, the whole resulting in an increase of about one hundred pages.

Assuming that the main efforts of the practitioner are to find out what is the matter with the patient and then to alleviate or cure, the sections on treatment are very complete and contain about 400 prescriptions.

The inclusion of "Diseases of the Skin," a subject generally omitted from works on practice, has gone far towards making the great popularity of the book. The same may be said of the shorter article on "Mental Diseases."

Dr. R. J. E. Scott, the editor, has had a wide literary and medical experience. He is well known by his contributions to the *New York Medical Record* on State board examinations, a subject with which he has become acquainted by reason of having made it a special study in connection with his teaching work and the preparation of the "State Board Examination Series."

---

PLASTER OF PARIS AND HOW TO USE IT.—By Martin W. Ware, M. D, New York; Adjunct Attending Surgeon, Mount Sinai Hospital; Surgeon to the Good Samaritan Dispensary; Instructor of Surgery in the New York Post-Graduate School. Second edition, revised and enlarged. Price, cloth, square form, $1.25; De Luxe leather, $2.50. Surgery Publishing Co., New York.

The exhaustion of the first edition and the persistent demand for this helpful book were the incentives for this second edition, which has been completely rewritten and enlarged, and thus its scope of usefulness has been greatly extended. Complete new drawings and marginal side notes in red embellish the book, and ninety illustrations are used to more clearly put up to the eye of the reader the intent of its subject matter.

Such information as History, Materials, Manufacture of Bandages, Storage, Bandages of Commerce, Calot Plaster Bandages, the Immediate Preparation of Bandages, Application and Precaution, Removal of Bandages, etc., are all given under the contents of the Plaster of Paris Bandages. Then follows such chapters are Application of the Plaster of Paris Bandage to Individual Fracture, Fractures of the Upper Extremity, Fractures of the Lower Extremity, Moulded Plaster of Paris Splints, Plaster of Paris in Orthopedic Surgery, etc., and all presented in such a comprehensive manner as to make this book of particular service to every doctor. The mechanical features of the book are decidedly striking.

Fun in a Doctor's Life—Being the Adventures of an American Don Quixote in Helping to Make the World Better, and How the Problem Was Solved for Him By Others, in England, France and Germany; Also an Endeavor to Convert What is Usually Stupid, Egotistical and Uninteresting in an Autobiography Into Many Short, Readable Stories and Essays; Didactic Only Through Entertaining. By Shobal Vail Clevenger, M. D., author of Medical Jurisprudence of Insanity, Evolution of Man and His Mind, Spinal Concussion, Therapeutics, Materia Medica and Practice, Artistic Anatomy, Method of Government Surveying, etc. 1909. Evolution Publishing Company, Atlantic City, N. J.

Quite a readable little book, by a seemingly very old gentleman. I did not see much fun in it, however.

---

In nephrectomy for hypernophroma, it is very important to remove the renal vein.—*American Journal of Surgery.*

